# Introduction and feeding practices of solid food in preterm infants born in Salzburg!

**DOI:** 10.1186/s12887-021-02505-6

**Published:** 2021-01-27

**Authors:** Edda Hofstätter, Verena Köttstorfer, Patricia Stroicz, Sebastian Schütz, Lorenz Auer-Hackenberg, Johannes Brandner, Martin Wald

**Affiliations:** 1 Division of Neonatology, Department of Paediatrics and Adolescent Medicine, Paracelsus Medical University, Salzburg, Austria; 2 Department of Mathematics, Paris Lodron University, Salzburg, Austria

**Keywords:** Complementary feeding, Preterm infants, Solids, Weaning

## Abstract

**Background:**

It is shown that meeting the increased nutritional demand of preterm infants from birth is not only important for survival but essentially contributes to the infants` overall development and long-term health.

While there are established guidelines for weaning term infants, evidence regarding preterm infants is scarce and less precise.

The aim of this study was to identify the current practices on introducing solids to preterm infants amongst caregivers in Salzburg and determine potential reasons for early weaning.

**Methods:**

Altogether 68 infants born between 24 0/7 and 36 6/7 weeks were recruited and detailed structured interviews with the caregivers were conducted at 17 weeks corrected age. Weight, height and head circumference were collected.

**Results:**

52% of the study group received solids before the recommended 17 weeks corrected age. For this group the mean age being 13.77 ± 1.11 weeks corrected age. Premature introduction of solids significantly correlates with exclusively and early formula-feeding. 34% were weaned due to recommendation by their paediatrician. 23% of the preterm infants even received solids before 12 weeks corrected age, putting them at risks for developing obesity, celiac disease and diabetes.

**Conclusions:**

This study shows the necessity for clear guidelines regarding the introduction of complementary feeding in preterm infants as well as the importance of their implementation. Caregivers should receive information on this topic early enough and they should fully understand the difference between chronological and corrected age.

**Supplementary Information:**

The online version contains supplementary material available at 10.1186/s12887-021-02505-6.

## Background

The probability of survival in preterm infants, especially in extreme premature babies born before 28 weeks of gestation has extended over the last few years due to great advances in both obstetric and neonatology procedures. Whilst in 1990 only a few babies born before 25 weeks of gestation had a chance of survival [1], by 2018 about half of the babies born this early survived [2].

Next to improving prenatal care, ventilation strategies and the introduction of advanced techniques in neonatal acute management, optimising nutrition led to dropping mortality and morbidity rates in these very vulnerable infants.

Preterm infants have increased nutritional needs and studies show that meeting those increased demands in calories, proteins, minerals and vitamins from the very beginning is not only important for the children’s survival but essentially contributes to the infants’ overall development and long-term health [3].

There have been international accepted guidelines regarding the optimal early parenteral and enteral nutrition in extremely preterm infants during their hospitalisation since the mid-90s. Special preterm formulas as well as human milk fortifiers are available and of high-quality for feeding preterm infants from birth until their hospital discharge. As of 2010 there are also post-discharge formulas available in Austria which are developed to meet the premature infant’s nutritional needs and help with catch-up growth after hospital discharge.

A recent commentary shows the significance of adequate parenteral and enteral nutrition as well as appropriate growth in this period and the need of further investigations [4].

A special group of preterm infants are late preterm infants. Almost 70% of all premature born infants are “late preterms” with a gestational age of 34/0–36/6 weeks. Because of their birth weight being usually above 2000 g, they are often regarded to as “almost term”. Often these preterm infants do not receive fortifiers or special preterm formula. In fact, these children are at higher risk to develop obesity in later life [5]. Despite their higher needs for food supporting adequate growth, they received low energy and / or low-protein dense foods first in complementary feeding [6].

In 2012 the Austrian Society for Paediatric and Adolescent Medicine (ÖGKJ) has modified the current guidelines of the European Society for Paediatric Gastroenterology, Hepatology and Nutrition (ESPGHAN) [7] and developed a position paper on feeding preterm infants post-discharge for Austrian neonatal outpatient clinics as well as family paediatricians to use in the post-discharge care of preterm infants. The aim of this paper was to support an optimal catch-up growth in preterm infants while avoid overfeeding to prevent metabolic disease in later life [8].

Whilst there are many studies on preterm nutrition regarding breast- and/or formula-feeding, the weaning period in preterm infants has attracted very little attention yet, despite the fact that solid foods will provide the main amount of macro-, und micronutrients from the time on when breastmilk or formula cannot longer meet a child’s nutritional needs.

Within the last few years the emphasis on infant nutrition, including complementary feeding has shifted from only prevention of malnutrition and promoting appropriate growth toward a well-balanced diet, facilitating long-term health benefits while preventing the risk of chronic disease. Studies show, that rapid weight gain during infancy puts children at higher risk of suffering from cardiovascular disease in adulthood [9], also untimely introduction of solids has been associated with an increased risk of obesity [10] and diabetes [11].

While there are some guidelines and recommendations regarding the timing of weaning particularly for term infants, by now [7], evidence on complementary feeding for preterm infants is still scarce and so far there are no distinct guidelines [12]. The current recommendations in Austria are based on the European guidelines and advise to use the infant’s corrected age and take neurodevelopmental progress as well as growth into consideration [8].

Previous studies on this subject suggest that preterm infants are introduced to complementary food significantly earlier than the recommended 17 weeks corrected age and also show that different factors like the mothers educational level or the infant’s gestational age at birth had an impact on the weaning behaviour [13, 14].

The aim of this study was to assess the current feeding practices amongst caregivers of preterm infants born in the Landeskrankenhaus Salzburg (Austria), including determining those factors that may influence the introduction of solids.

## Methods

### Participants

Ethical consent has been obtained from the responsible local research Ethical Committee.

100 premature infants born at Salzburg university medical hospital reached a corrected age of 17 weeks in a 4-month study-period, ranged from 24 0/7 weeks to 36 5/7 weeks gestational age.

All parents who agreed to participate in the study provided informed consent.

Eligibility criteria included prematurity and at least one of the parents had to be fluent in either German or English.

Exclusion criteria included surgical problems that interfered with normal nutritional behaviour (e.g. gastroschisis or oesophageal atresia), genetic syndromes, metabolic diseases and mothers who suffered from an untreated psychiatric illness as well as children who were placed in foster care. In addition, an extremely prolonged necessity for gavage feeding or the recurrent need of a feeding tube around the time of inquiry was an exclusion criterion.

### Questionnaires

To prevent biasing the study by raising extraordinary awareness to complementary feeding and its guidelines, all families were contacted as early as 16 weeks corrected age to minimise recall bias regarding the time of weaning and which food groups they were first introduced to, carers were contacted at the latest of 18 weeks corrected age.

Detailed structured interviews were administered either via telephone or in person in the outpatient clinic.

Collected data included sociodemographic data like information on the infant’s family situation, particularly information concerning the mothers, like maternal age, special diet and potential diseases before and during gestation, previous pregnancies and births, as well as educational status and family income [Table 6, supplemental file Questionnaire 1].

Perinatal data contained gestational age, birth weight, birth height and head circumference at birth, along with gender, whether it was a singleton or multiple pregnancy, birth mode, 1,5, and 10-min APGAR (Appearance, Pulse, Grimace, Activity, Respiration) and relevant medical problems and interventions during or immediately after birth [Table 6, supplemental file Questionnaire 1].

### Feeding practices

Information regarding the infant’s feeding practices from birth were obtained, including detailed information on milk-feeding practices. It was documented whether the infants have been breastfed or formula-fed right after birth, if they still were breast-fed at the time the survey was conducted and if so, if they were still fully or partially breastfed along with solids and/or formula. Duration of exclusively vs. partially breastfeeding was documented both from birth and term.

Since all our participants were born preterm, information on fortifying breastmilk or feeding special preterm formula regarding the duration and type of supplementation also have been obtained.

It was documented whether the infants were already started on solids or not, and if so the corrected age as well as the uncorrected age of the complementary food introduction was noted in weeks. All mothers provided us with information about when they introduced specific food groups to their children, which were again recorded in weeks from birth and term.

Foods or food groups of interest included vegetables, potatoes, meats, cereal, cereal-milk purees, fruits, eggs, fish, water, tea and sweetened beverages.

Information was collected on how often children were introduced to new foods within 1 week and whether they received a special diet (for example vegetarian).

Mothers also provided information about personal reasons on why they started weaning their infants and whether they specifically sought information on this topic like talking to their paediatrician, reading on-topic books, brochures etc. [Table 7, supplemental file Questionnaire 2].

### Measurements

Weight, height and head circumference at birth and at the time of the interview were collected, as well as those of previous outpatient-clinic visits.

All measurement-data has either been gathered from medical reports of our department during in- and outpatient visits, or measured by paediatricians and were provided to us by the infant’s mothers.

Weight and height data until a gestational age of 50 weeks were scaled based on the gender-specific Fenton Preterm Growth Charts [15].

Measurements taken as of 51 weeks gestational age were scaled based on the gender-specific WHO growth charts *“weight for age, birth to 6 months”* and *“height for age, birth to 6 months”* using the infant’s corrected age.

All weight measurements, whether obtained in the clinic or those taken by family paediatricians were taken on calibrated baby-scales. All height measurements were taken by measuring the infant in a supine position from the top of the head to heel, with the infant’s leg being stretched out on a flat surface.

### Statistical analysis

Data consistency was checked and data were screened for outliers and normality by using quantile plots. Crosstabulation tables with Fisher’s Exact test or Pearson’s test were used to analyze crosstabulations. Two-sided Student t-tests with and without the assumption of variance homogeneity were used to compare expectation values among different groups and 95% confidence intervals were used estimate the effects. Kaplan-Meier analyses were done and survival curves were tested using Cox-F and Gehan’s and Wilcoxon test. All reported tests were two-sided, and *p*-values < 0.05 were considered as statistically significant. All statistical analyses in this report were performed by use of NCSS (NCSS 10, NCSS, LLC. Kaysville, UT), Mathematica 7 (Wolfram Research, Inc., Mathematica, Version 7.0, Champaign, IL), STATISTICA 13 (Hill, T. & Lewicki, P. Statistics: Methods and Applications. StatSoft, Tulsa, OK).

## Results

### Study population

**Fig. 1:**
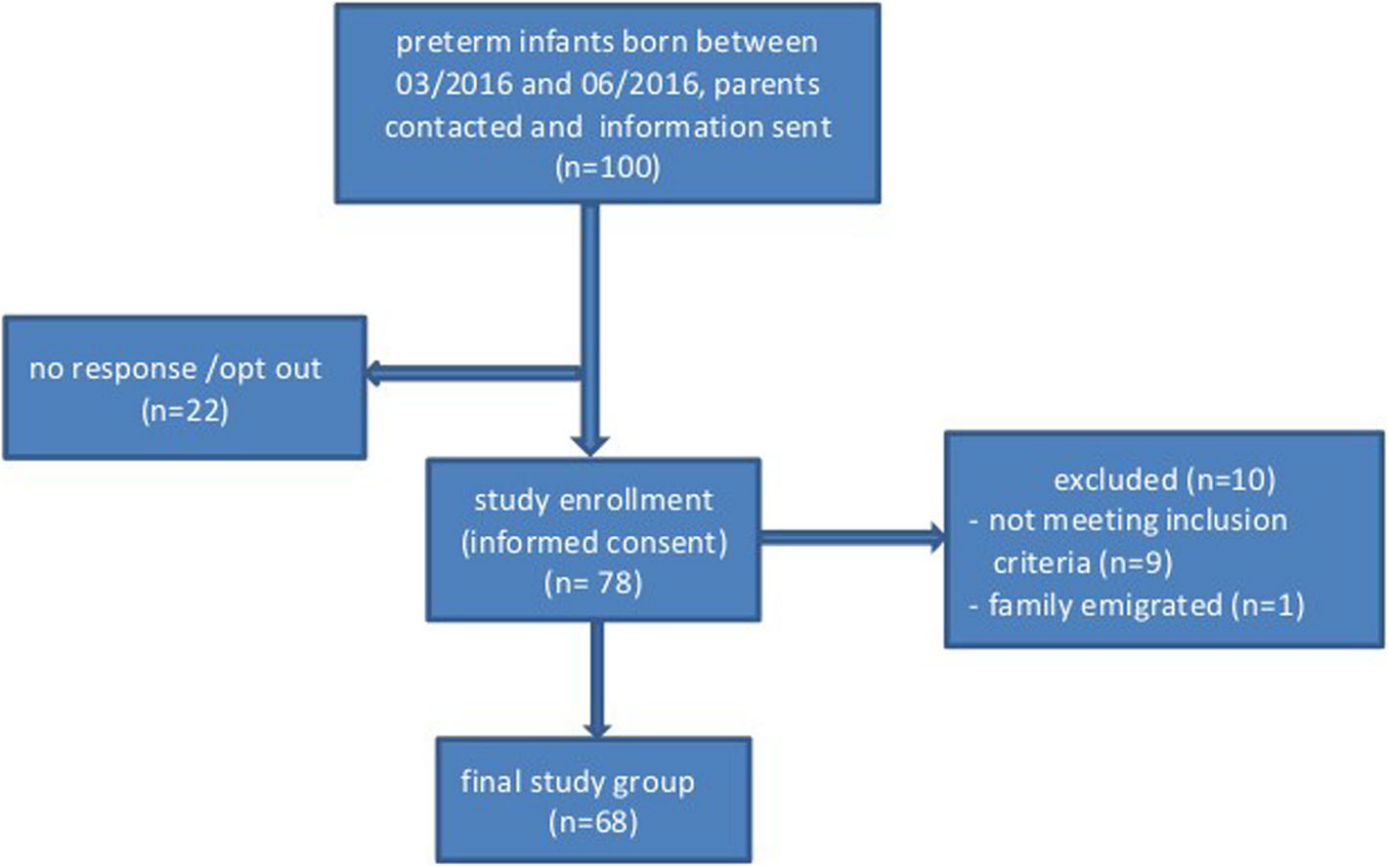
Flow chart of search strategy and selection process

An overall of 68 infants were successfully recruited into the study. Flow chart of search strategy and selection process is shown in Fig. 1 and basis data of the study population are listed in Table 1.

**Table 1:** Description of study population

***n*** = 68	Mean	SD
Gestational age (weeks)	34,1	2,53
Birth weight (g)	2212,1	585,46
Birth length (cm)	45,3	4,04
Birth head circumference (cm)	31,4	2,59
	**%**	**n**
Female	40	27
Male	60	41
Vaginal delivery	40	27
Caesarean delivery	59	40
Assisted delivery	1,5	1
Singleton	71	48
Twins	29	20
SGA <10th percentile	8,8	6
SGA <3^rd^percentile	7,4	5
< 28 weeks of gestation	2,9	2
28/0–31/6 weeks of gestation	10,3	7
> 32/0 weeks of gestation	87	59

52% of the study group were started on complementary feeding before the recommended 17 weeks corrected age. The 95% confidence interval ranges from 34 to 64%. This group will be referred to as *early weaning group* in the following. The remaining 48% of infants who received solids at the earliest of 17 weeks corrected age will be referred to as the appropriate weaning group.

### Socio-demographic characteristics

Infants were recruited from a cross-section of socio-economic groups. All but four infants were of Caucasian ethnic origin.

In Table 2, the socio-demographic and socio-economic characteristics between the two groups are compared.

**Table 2:** Comparison of maternal socio-demographic and socio-economic characteristics

	weaned < 17 weeks ***n*** = 35 (52%)	weaned > 17 weeks ***n*** = 33 (48%)	
	***Mean ± SD***	***Mean ± SD***	***p-value***
Maternal age	31,8 ± 5,26	31,27 ± 5,67	0,691
Gravidity	2,29 ± 1,54	1,61 ± 0,96	0,034
Parity	1,77 ± 0,97	1,36 ± 0,70	0,051
Pre-pregnancy BMI (kg/m^2^)	24,63 ± 4,15	24,21 ± 4,49	0,69
Children <18y living in the same household	2,11 ± 0,9	1,52 ± 0,67	0,003
	***n = (%)***	***n = (%)***	***p-value***
Gestational diabetes (diet)	*n* = 7 (20)	*n* = 0 (0)	0,011
Gestational diabetes (insulin)	*n =* 3 (8,6)	*n* = 2 (6,1)	1
Preeclampsia	*n =* 2 (5,7)	*n* = 4 (12,1)	0,421
HELLP	*n =* 3 (8,6)	*n =* 1 (3,0)	0,614
Hypertonia (during pregnancy)	*n =* 4 (11,4)	*n =* 2 (6,1)	0,674
Maternal educational level			
8 to 10 Years (High School Equivalent)	*n* = 16 (45,7)	*n* = 13 (39,4)	0,632
10 to 12 years (General qualification for university entrance)	*n =* 7 (20,0)	*n* = 8 (24,2)	0,773
12 to 14 years (College Equivalent)	*n* = 5 (14,3)	*n =* 4 (12,1)	1
> 14 years of education (University)	*n =* 7 (20,0)	*n =* 8 (24,2)	0,773
Household income			
< €30.000	*n =* 16 (47,1)	*n =* 5 (15,2)	0,009
€30.000–60.000	*n =* 17 (48,6)	*n* = 25 (75,8)	0,03
> €60.000	*n =* 1 (2,9)	*n =* 3 (9,1)	0,35

In the following passage, you can find the maternal and family characteristics. The mean age of mothers in the study was 31.54 years that did not differ significantly in the two groups.

Infants born into families with more children were introduced to solids significantly earlier than those with fewer siblings (*p* < 0,01). Mothers who experienced more pregnancies overall started introducing solids earlier than those with less pregnancies (*p <* 0,05).

It was noticeable that 20% of mothers in the early weaning group had developed non-insulin dependent gestational diabetes compared to 0% in the appropriate weaning group, which shows a significant difference. Other health problems during the pregnancy like HELLP syndrome (haemolysis, elevated liver enzymes, and low platelet count), preeclampsia or hypertonia showed no significant differences between the two groups.

Another factor significantly associated with earlier complementary food introduction was lower household income, infants of families who lived on less than 30.000€/year received solids significantly earlier (*p* < 0,01).

Table 3 shows the infant characteristics and differences between the early and the appropriate weaning group. Regarding the measurements at birth only the head circumference was significant. Infants in the early weaning group had a significantly wider head circumference at birth than those in the appropriate weaning group.

**Table 3:** Comparison of infant characteristics between the early and appropriate weaning group

	weaned < 17 weeks ***n =*** 35 (52%)	weaned > 17 weeks ***n =*** 33 (48%)	
	**Mean ± SD**	**Mean ± SD**	***p-*value**
Gestational age (weeks)	34,43 ± 1,51	33,77 ± 3,28	0,286
Birthweight in grams	2325,17 ± 522,12	2092,24 ± 631,84	0,101
Birth length in cm	46,01 ± 3,24	44,61 ± 4,69	0,155
Birth head circumference in cm	32,04 ± 2,14	30,68 ± 2,87	0,03
Birth weight percentile	52,77 ± 29,55	44,15 ± 25,96	0,207
Birth length percentile	63,20 ± 30,03	59,21 ± 25,12	0,556
Birth head circumference percentile	64,94 ± 26,61	53,33 ± 29,83	0,095
	***n =*** **(%)**	***n =*** **(%)**	***p-*value**
Females	*n =* 13 (37,1)	*n* = 14 (42,4)	0,805
Males	*n* = 22 (62,9)	*n* = 19 (57,6)	0,805
Singletons	*n =* 19 (54,3)	*n* = 29 (87,9)	0,003
Twins	*n =* 16 (45,7)	*n =* 4 (12,1)	0,003

In addition, measurements at 17 weeks corrected age showed no significant differences in the two groups except for weight percentile, which was again significantly higher in the early weaning group. Although the infant’s gender did not have any effect on the timing of complementary food introduction, there were significantly more twins in the early weaning group (*p* < 0,01).

Furthermore, we present the different feeding practices.

Milk feeding practices and their differences between the two weaning groups are represented in Tables 4 and 5. Almost 95% of the entire study group were exclusively breastfed, when divided in the two weaning groups. 100% were ever breastfed in the appropriate weaning group and almost 90% in the early weaning group.

**Table 4:** Compared milk-feeding practices of the two weaning groups

	weaned < 17 weeks ***n =*** 35 (51.5%)	weaned ≥ 17 weeks ***n =*** 33 (48.5%)	
	***n =*** **(%)**	***n =*** **(%)**	***p-*value**
Ever breastfed	*n =* 31 (88.6)	*n =* 33 (100)	ns
Exclusively breastfed at 17 weeks corr.age	*n =* (0)	*n =* 15 (45.5)	0
Partially breastfed +formula at 17 weeks corr.age	*n =* 4 (11.4)	*n =* 6 (18.2)	ns
Partially breastfed +solids at 17 weeks corr.age	*n =* 8 (22.9)	*n =* 2 (6.1)	ns
Partially breastfed +solids and formula at 17 weeks corr. Age	*n =* 2 (5.7)	*n =* 0 (0)	ns
Completely stopped breastfeeding before 17 weeks corr.age	*n* = 21 (60)	*n* = 10 (30.3)	0.017
Ever formula-fed	*n =* 31 (88.6)	*n =* 16 (48.5)	0.001
Ever received preterm formula	*N =* 17 (48.6)	*N =* 8 (24.2)	0.047

**Table 5:** Milk feeding practices of the entire study cohort

Study cohort ***n =*** 68 (100%)	***n =*** (%)
Ever breastfed	*n* = 64 (94)
Exclusively breastfed at 17 weeks corr. Age	*n =* 15 (22)
Partially breastfed + formula at 17 weeks corr. Age	*n* = 12 (18)
Partially breastfed + solids at 17 weeks corr. Age	*n* = 12 (18)
Partially breastfed +solids and formula at 17 weeks corr. Age	*n =* 2 (6)
Completely stopped breastfeeding before 17 weeks corr. Age	*n =* 29 (43)
Ever formula-fed	*n =* 47 (69)
Ever received preterm formula	*n =* 25 (37)

60% of all infants in the early weaning group completely stopped breastfeeding before 17 weeks corrected age, compared to 30% in the appropriate weaning group, which was significant (*p* < 0.05). 70% in the appropriate weaning group still received breastmilk at 17 weeks corrected age, as opposed to 40% in the early weaning group. Almost 50% of the infants in the appropriate weaning group were still exclusively breastfed at the time of the inquiry.

70% of all infants exclusively received formula; almost 40% were fed special preterm-formula. In the early weaning group, 90% of the infants were formula-fed, in the appropriate weaning group it was only 50%, which was highly significant (*p* < 0,01).

**Fig. 2:**
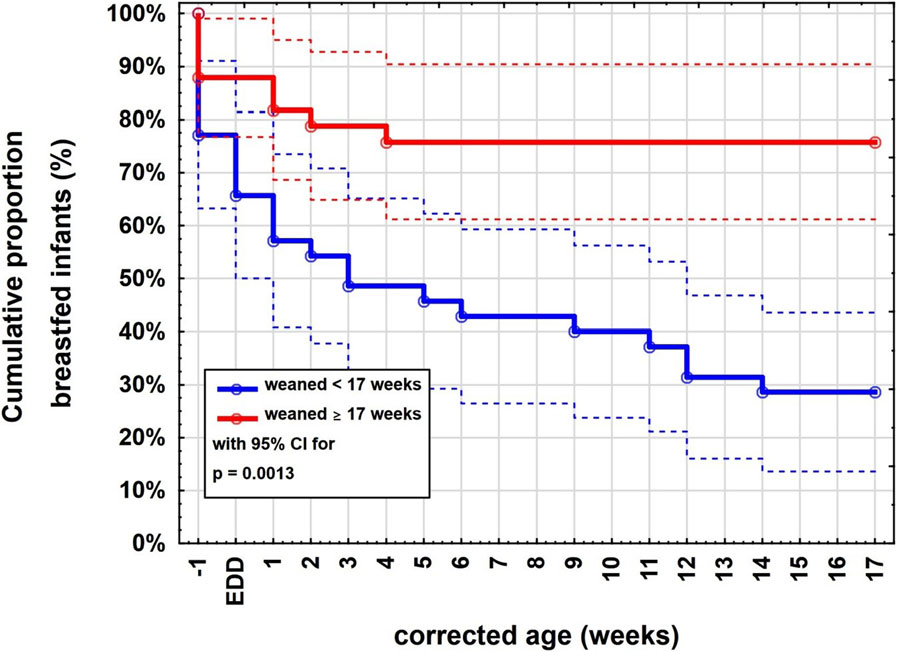
Cumulative proportion of exclusively and partially breastfed infants

In Fig. 2 you can see that the proportion of breastfed infants in the early weaning group is a bit smaller from the beginning and is dropping very rapidly to under 50% at around 4 weeks corrected age and keeps dropping. In the appropriate weaning group breastfeeding rates drop in the first few weeks and then stabilize at about 70%.

**Fig. 3:**
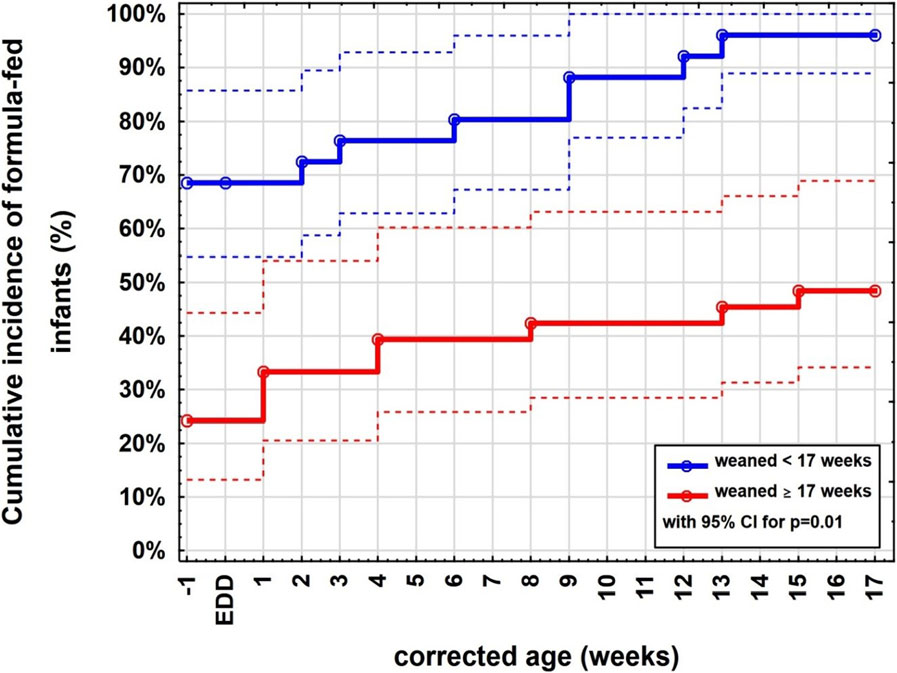
Cumulative incidence of formula-fed infants

In Fig. 3, it is shown that the proportion of formula-fed infants in the early weaning group is significantly higher from birth than in the appropriate weaning group and is increasing to almost 100% until 17 weeks corrected age opposed to the appropriate weaning group with 50% receiving formula.

**Fig. 4:**
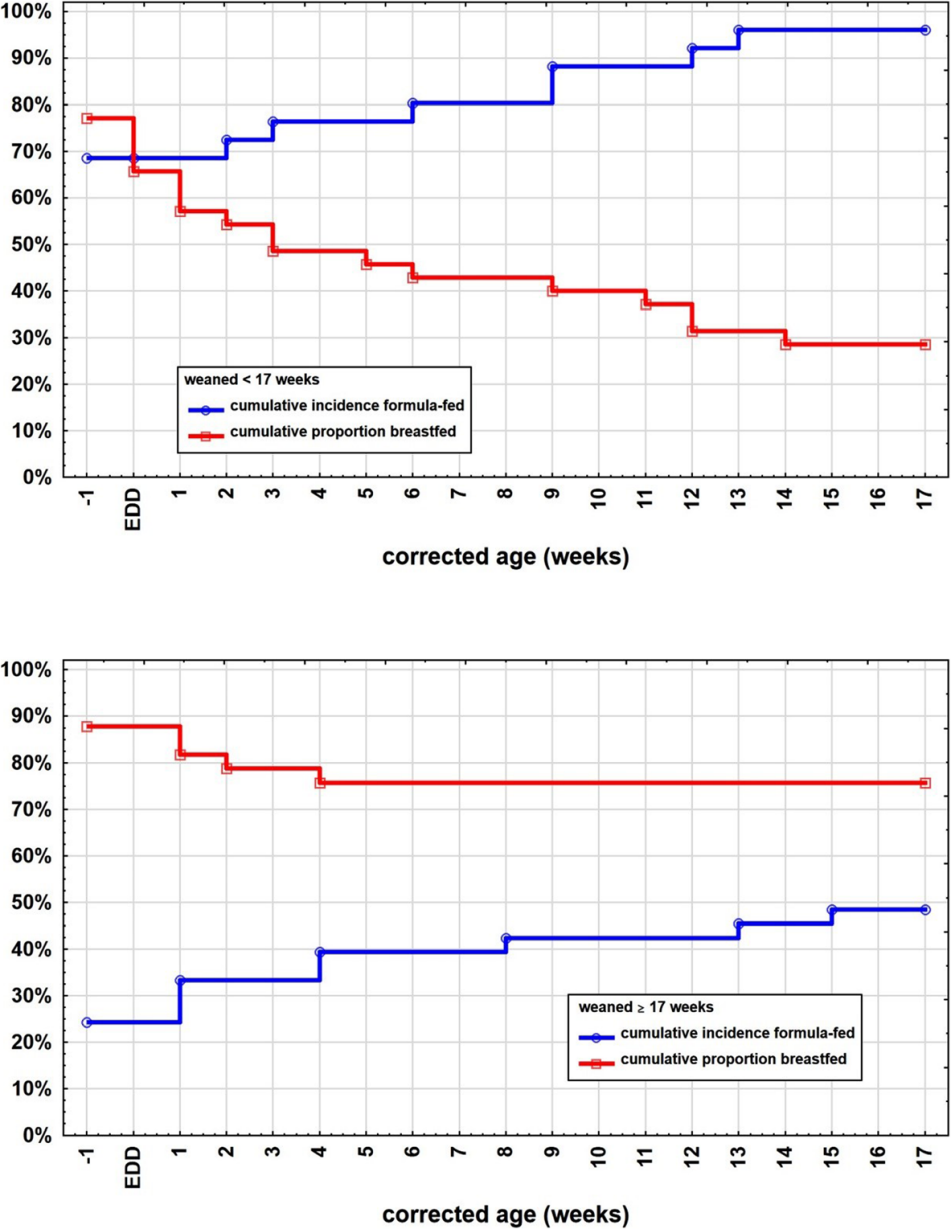
Graph showing the difference between the correlation of breastfeeding and formula-feeding in the two weaning groups

The correlation between breastfeeding and formula feeding between the two weaning groups is diagrammed in Fig. 4. In the early weaning group, breastfeeding proportions are rapidly dropping whilst the proportion of formula feeding increases rather quickly. In contrast, breastfeeding in the appropriate weaning group also decreases but only at a fraction of the other weaning group’s percentage and much slower. Also the formula-feeding proportion does not increase as fast and as much as in the early weaning group, indicating that infants who get introduced to solids untimely are also much more likely to be formula-fed than breastfed.

Information on complementary feeding has only been obtained from the early weaning group in this study and is summed up in Table 6 (supplemental file Questionnaire 1).

An overall of 35 infants received solids prematurely, starting at the earliest of 10 weeks corrected age. 89% of those infants received vegetable-puree as their first complementary food, and the remaining 11% were offered fruit-puree as their first weaning food. 31% did still only receive vegetable- and or fruit-puree at 17 weeks corrected age.

Only 26% of the infants ever received meat by the time of the survey, 9% were offered fish. 37% were offered unsweetened drinks, mostly water, on a regular basis, 6% received sugared drinks like fruit juice or other sweetened drinks.

26% of the mothers introduced a new food to their infant daily, 31% every 2–3 days, 17% every 4–5 days, 23% once a week and 3% less than once a week.

Most of the mothers obtained information about weaning their premature infants from their paediatrician, almost all scheduled an appointment at the paediatricians practice, and only two got their information by phone. 28% already had weaning experiences with older children and respectively 17% obtained information from booklets and the internet.

Almost half of the mothers stated their infants clearly showed interest in eating as their main reason to start weaning, 9% felt pressured by their families and therefore introduced solids early, another 9% of the mothers stated that they wanted their children to be able to eat solids so they could stop breastfeeding sooner and therefore started weaning.

34% stated that their paediatrician recommended starting complementary feeding.

## Discussion

The results of this study exhibit, more than half (52%) of the preterm infants in this cohort were introduced to complementary food before 17 weeks corrected age as recommended by the ÖGKJ [8]. The present study’s findings are consistent with previous international research showing that a significant proportion of premature born infants receive their first solid food earlier than recommended [13]. Actually 23% of the preterm infants who were given solids early in Salzburg received complementary food even before 12 weeks corrected age, putting them at risk for Diabetes Mellitus (DM) and coeliac disease as studies show [10]. The risk for developing obesity is discussed controversially [16].

Previous research has identified predictors of early weaning in preterm infants including male sex, gestational age, younger maternal age, maternal smoking, lower level of maternal education, higher maternal pre-pregnancy BMI and formula-feeding [13].

Results of this study support the finding that formula-feeding increases the odds for an early introduction of complementary feeding. Also symptomatically for that are mothers with non-insulin dependent gestational diabetes. All these mothers in our study started weaning their infants before the corrected age of 17 weeks. Mothers suffering from gestational diabetes usually tend to come into lactation more slowly and to have less breastmilk than healthy mothers do, which often leads to early formula feeding. Just as the number of pregnancies, the number of children living in the same household as well as twins being a predictor we think this may be explained by the mother’s lack of time, possible due to lack of supportive help. Furthermore, having more children to take care of might leave mothers too stressed to keep up breastfeeding for a prolonged period of time, so they start formula-feeding only a few weeks postpartum which leads them to be prone to earlier solid food introduction. Typically, formula fed infants with higher weight percentile at 17 weeks corrected age are weaned early. Additionally, a lower household income is also associated with earlier weaning. It might be speculated that a lower household income is associated with a lower level of maternal education and “normal” family food is cheaper than buying formula. A direct correlation of a lower level of maternal education is not found. As well younger maternal age, higher maternal pre-pregnancy BMI, male sex and gestational age are also not correlated in our study. This may be due to the small sample size. Information on smoking habits was not obtained.

In adherence to data from term and preterm infants [13] the present study also confirms that exclusive and prolonged breastfeeding is positively correlated with an appropriate introduction of complementary feeding. Mothers who tend to exclusively breastfeed over a longer period of time were also less likely to stop breastfeeding abrupt when introducing solids, but kept on partially breastfeeding for as long as possible, enabling their infants to profit from human milk.

during their weaning period and further on. In comparison, mothers who decided on combined milk feeding during the infant’s first weeks of life tended to completely stop breastfeeding very soon after starting formula-feeding and those mothers were also found to start feeding solids early.

An additional key aspect of this study was to identify the types of food given to preterm infants mainly during the first few weeks of complementary feeding. Again, this study can only provide data on those aspects from the early weaning group.

There is international consensus that from time complementary foods is started, relatively fast – within 2 to 3 weeks complex food containing high energy density, proteins and minerals like iron and zinc should be introduced [7]. It is also shown that the use of foods with a higher energy and protein content as well as foods that are rich in iron and zinc have positive effects on the increase in standard deviation length scores and the length growth velocity. These children also have higher haemoglobin and serum iron levels at the age of 6-month [17].

Previous research found international discrepancies on weaning foods, particularly on the first solid foods given. While almost 85% of British preterm infants received baby rice as their first complementary food and 6% received fruit or vegetables [13], in Italy almost half of the preterm infants received mashed fruit as their first solids [18]. Fanaro et al. expressed his concern about Italian preterm infants being weaned with low energy and low nutrient-dense food. Meat as part of early solid feeding was offered to only about 10% of infants in the Italian study [18]. For Austrian infants the ÖGKJ recommends starting complementary feeding with bioavailable iron and zinc sources like meat and grains, otherwise it is not mandatory to follow a specific order of food introduction [8]. Those recommendations seem to be very clear and easy to implement. In the present study third of infants in the early weaning group still received only fruits and vegetables at 17 weeks corrected age, although some of them had been weaned as early as 10 weeks corrected age. Almost 90% of preterm infants received vegetable puree as first solid food, the remaining 10% were started on solids with fruit puree. About 25% received a form of milk-free cereal within their first week of complementary feeding; meat was introduced to about 20% of infants in the first week. When mothers were asked about reasons for choosing vegetables or fruit as first solid food the main proportion reported vegetables being recommended by their midwife as best. There seemed to be awareness amongst mothers on nutritional value of foods to some extent, as most of them chose not to introduce their infants to fruits during their first week of weaning because they didn’t want them to get used to foods with high sugar content to soon. Some mothers admitted to buy commercially available baby-food jars because they felt insecure on what type of food to feed their infants. Furthermore, they relied on the labelling of the food being age appropriate. If asked about introducing meat, some mothers reported they felt their infants being too small to be fed anything else besides vegetables and fruits. Some mothers reported they felt their infants did not enjoy eating solids and therefore they did not want to overstrain them by introducing too many different types of food.

When asked about if and where they obtained information on introducing complementary feeding to their infant, about half of mothers reported talking with their family paediatrician about introducing solids, most of them during one of their infant routine check-ups, some called or made an extra appointment. Many mothers felt insecure about how and when to wean their premature born infant, even if they already had older children, which is why they set high value on the family paediatrician’s recommendations.

As already mentioned above, this study identified detailed information on caregiver’s reasons for weaning their preterm infant early. More than one third of mothers reported their infant showed increased interest in food during family meal times and they felt breastmilk respectively formula did not satisfy their infant any longer. It is possible that mothers over interpreted those signs of dissatisfaction as infants at this age naturally start putting their hands and toys into their mouths and showing more and more interest to their environment which represents only a developmental step without necessarily expressing readiness for complementary feeding. It is stated in other studies that one important factor, before weaning children, is that they have a good head control in terms of an adequate neurological muscular development and can sit in an upright position [19]. Another third of mothers started introducing solids to their preterm infants because their family paediatrician recommended it to them. We do not have a definite explanation but some theories as to why paediatricians would recommend early solid introduction to preterm infant’s caregivers. Premature infants represent only a small group of infants who are roughly uniformly distributed to all family paediatricians in Salzburg and its surrounding areas, hence premature born infants are only a fraction of a family paediatrician’s patient clientele. As there are neither distinct guidelines nor much research on solid food introduction for this specific but yet small group of patients, a good proportion of family paediatricians just might not know about the current recommendations. In addition, most of infants in this study cohort were late preterm infants with little to no long-term health problems at the age of solid food introduction, which could lead to paediatricians overlooking their prematurity when giving complementary feeding recommendations. Supporting this theory would be our finding that when conducting the interviews, it was noticed that especially mothers of late preterm infants who only spent a few days in the NICU (neonatal intensive care unit) or didn’t need any monitoring at all had often forgotten about their child being born prematurely respectively considered it irrelevant by then.

Nearly 10% of mothers felt pressured by their family and close friends to start introducing solids, which can be explained by unawareness of prematurity as well as absent knowledge and understanding of current recommendations and corrected age. About 6% of mothers reported introducing complementary feeding because their infants were 4 months uncorrected age, which supports the assumption that the term “corrected age” is not something all caregivers fully understand. If the data are considered from birth, preterm infants in the present study were weaned with 19.37 weeks ±2.31 weeks chronological age. If that was the case, mothers would very well follow the current guidelines on weaning their infants and missing compliance would not be as big a problem as previous studies suggest [13].

To our knowledge, this study is the first to explore complementary feeding practices in Austria and there is no similar national data on preterm infants. The classification “preterm” includes a very heterogeneous group with a wide range of gestational age and birth weight; however, most of the infants will be developmentally challenged to some extent, for example poor sucking reflex, reduced respiratory capacities.

One major strength of this study is its prospective design. Because the questionnaires were conducted at exactly 17 weeks corrected age with a maximal variances of 1 week before or after, recall bias regarding the time of solid food introduction, types of food or reasons for starting complementary feeding is nearly impossible.

There are studies about different factors that affect the introduction of complementary foods in preterms e.g. earlier weaning in formula-fed infants [13]. However, to the best of our knowledge there is no other study obtaining detailed information from caregivers on their reasons to start weaning their preterm infants. This information seems to be very important to prevent too early introduction of solids in the future. Finally, although the cohort is rather small, it still gives a good insight on the weaning practices of Austrian preterm-caregivers and is, to our knowledge, the first national study on this.

There are a few limitations to the present study. The preterm cohort studied in Salzburg was self-selected and is not representative of the preterm population in general. The rather small study cohort is due to a very limited time frame for recruiting families and because we only recruited infants from Salzburg. Therefore, only assumptions can be made regarding the weaning practices for the rest of Austria. Mean maternal age in this study was 31.54 y ± 5.43, that is slightly older than the mean maternal age of 30.5y in Austria (data obtained from the 2015 report of Austrian Institute for Family Studies). Regarding the types of complementary food given there is only data from the 52% of preterm infants who received solids before 17 weeks corrected age, which was the time of our questionnaire being obtained, as we interviewed the families only once. Naturally, from the infants who had not yet received any solids at 17 weeks, any information on complementary feeding could not be obtained. Also reasons for introducing complementary feeding as well as sources of information on this topic could only be provided by families who had already started weaning their infants.

## Conclusions

The results of this study clearly highlight the need for practical guidelines for introducing complementary food to preterm infants. Those guidelines ideally will be based on the specific requirements of this heterogeneous group of infants, contributing to improved long-term health outcomes in an increasing number of premature born infants. For now, it is important to provide individual advice for parents, based on the infant’s gestational age at birth, nutritional requirements, medical problems and developmental status.

It would be reasonable for mothers of preterm infants to receive information on this topic during their NICU stay or during one of their outpatient follow-up visits. Furthermore, it would be beneficial to make sure caregivers fully understand the difference between chronological and corrected age. This could not just lead to a reduced incidence of early weaning but also might help mothers to explain their decision to delay introducing solids, hopefully reducing them feeling pressured by others.

Family paediatricians should be informed about current research and also be reminded that late preterm infants are still to be considered as premature born infants rather than almost term born infants, and therefore require special attention to some extent. This point is of particular importance if there are already term born infants in the family.

## Supplementary Information

**Additional file 1.**

**Additional file 2.**

## Data Availability

The datasets analysed during the current study are available from the corresponding author on reasonable request.

## Abbreviations

APGAR: Appearance, Pulse, Grimace, Activity, Respiration
BMI: Body mass index
DM: Diabetes mellitus
ESPGHAN: European Society of Paediatric Gastroenterology, Hepatology and Nutrition
HELLP: Haemolysis, elevated liver enzymes, low platelet count
NICU: Neonatal intensive care unit
ÖGKJ: Austrian Society for Paediatric and Adolescent Medicine
