# Supplementary material for: Introduction and feeding practices of solid food in preterm infants born in Salzburg!
Source: BMC Pediatr. 2021 Jan 27;21:56. doi: 10.1186/s12887-021-02505-6 (PMC7839190; doi:10.1186/s12887-021-02505-6)
Supplement: Supplementary file 1 — Additional file 1. [file 12887_2021_2505_MOESM1_ESM.pdf]

Peripartal information

mother's age at birth …….. gravidity / parity …….. / ……..

BMI (at beginning of pregnancy) …….. weight gain during pregnancy in kg …….

relevant previous illness (e.g. Diabetes mellitus, metabolic disease, …)

………………………………………………………………………………..

illness during pregnancy (e.g. pregnancy related diabetes)

…………………………………………………………………...

special diet of the mother before / during pregnancy:

before: □ none □ vegetarian / vegan □ other: ……..

during: □ none □ vegetarian / vegan □ other: ……..

marital status: □ single □ in a partnership

children < 18 years old living in the same household: ……..

the highest educational attainment: ……..

profession: ……..

nationality: mother: …….. father: ……..

native language: mother: …….. father: ……..

average annual family income (net)

□ < 30.000 € □ 30.000-60.000 € □ > 60.000 €

Perinatal information

gestational age: …….. (weeks) / …….. (days)

weight at birth: ……. g length at birth: ……. cm head circumference at birth: ……. cm

sex: □ female □ male pregnancy: □ single □ twin □ triplet

mode of delivery: □ vaginal delivery □ caesarean section □ vacuum □ other

APGAR 1 / 5 / 10 min. …….. / …….. / …….. cord arterial blood pH: …….

relevant medical problems during birth: …………………………………………………..
