# Supplementary material for: Introduction and feeding practices of solid food in preterm infants born in Salzburg!
Source: BMC Pediatr. 2021 Jan 27;21:56. doi: 10.1186/s12887-021-02505-6 (PMC7839190; doi:10.1186/s12887-021-02505-6)
Supplement: Supplementary file 2 — Additional file 2. [file 12887_2021_2505_MOESM2_ESM.pdf]

Indicate the actual body measurements of your child.

weight: ……. g   height: ……. cm   head circumference: ……. cm

percentile (WHO):   weight: …….   height: …….   head circumference: …….

Has / had your child any health problems? If yes, what kind of and when?

…………………………………………………………………………………………...

Do / did you breastfeed your child or do / did you offer breast milk?

□ Yes: □ breastfeed   □ breast milk   □ donor human milk

<u>at the moment:</u>   □ exclusively breastfeeding   □ partial breastfeeding

□ supplementary food   □ formula

<u>before:</u>   □ exclusively breastfeeding until …….. weeks of age

□ partial breastfeeding until …….. weeks of age

□ No, I never breastfeed / give breast milk

Did your child ever receive fortification of breast milk (e.g. FMS / FM 85)?

□ No   □ Yes: □ until calculated date of birth   □ until corrected 12 weeks of age   □ other

Did your child ever receive formulae for premature infants. (e.g. Aptamil PDF / Beba FG 1 or 2)?

□ No   □ Yes: □ until calculated date of birth   □ until corrected 12 weeks of age   □ other

What kind of formulae did your child receive?

□ None   □ PRE until ……. weeks of age

□ formulae for premature infants step 1 until ……. weeks of age

□ formulae for premature infants step 2 until ……. weeks of age

□ Formula 1 until ……. weeks of age   □ Formula 2 until ……. weeks of age

□ other: ……..

Have you started with supplementary food yet?

□ No □ Yes, since ……. weeks of age (corrected age …… in weeks)

When did you start the following foods?

vegetable gruel	□ ……. weeks of age	□ never
vegetable/grain	□ ……. weeks of age	□ never
meaty food	□ ……. weeks of age	□ never
grain porridge (without milk)	□ ……. weeks of age	□ never
grain porridge (with milk)	□ ……. weeks of age	□ never
fruit-based mash	□ ……. weeks of age	□ never
water / tea (sugarfree)	□ ……. weeks of age	□ never
tea (with sugar)	□ ……. weeks of age	□ never
fruit juice / sweetened drinks	□ ……. weeks of age	□ never
other (eggs, fisch)	□ ……. weeks of age	□ never

How often do you start a new sort of food?

□ every day □ every 2-3 days □ every 4-5 days □ once a week or less

Does your child receive a special diet at the moment e.g. vegetarian? If yes, what?

……...

Why did you start with supplementary food?

□ my child didn't have enough / was still hungry

□ my child was interested in / ready for food

□ my pediatrician recommended me to start with

□ other: ……..

How did you get information about supplementary food?

◻ conversation with pediatrician ◻ information booklet

◻ books: ……..

◻ „Baby led weaning“

◻ internet

◻ other: …….

Does your child get at the moment …

… supplemental iron: ◻ yes ◻ no

… supplemental vitamins: ◻ yes ◻ no
